# Cost of installing and operating an electronic clinical decision support system for maternal health care: case of Tanzania rural primary health centres

**DOI:** 10.1186/s12913-015-0780-9

**Published:** 2015-04-02

**Authors:** Happiness Pius Saronga, Maxwell Ayindenaba Dalaba, Hengjin Dong, Melkizedeck Leshabari, Rainer Sauerborn, Felix Sukums, Antje Blank, Jens Kaltschmidt, Svetla Loukanova

**Affiliations:** Behavioural Sciences Department, Muhimbili University of Health and Allied Sciences, Dar es Salaam, Tanzania; Navrongo Health Research Centre, Navrongo, Ghana; Centre for Health Policy Studies, Zhejiang University, Hangzhou, China; Institute of Public Health, University of Heidelberg, Heidelberg, Germany

**Keywords:** Cost analysis, Clinical decision support system, Maternal health, Rural area, Primary health center

## Abstract

**Background:**

Poor quality of care is among the causes of high maternal and newborn disease burden in Tanzania. Potential reason for poor quality of care is the existence of a “know-do gap” where by health workers do not perform to the best of their knowledge. An electronic clinical decision support system (CDSS) for maternal health care was piloted in six rural primary health centers of Tanzania to improve performance of health workers by facilitating adherence to World Health Organization (WHO) guidelines and ultimately improve quality of maternal health care. This study aimed at assessing the cost of installing and operating the system in the health centers.

**Methods:**

This retrospective study was conducted in Lindi, Tanzania. Costs incurred by the project were analyzed using Ingredients approach. These costs broadly included vehicle, computers, furniture, facility, CDSS software, transport, personnel, training, supplies and communication. These were grouped into installation and operation cost; recurrent and capital cost; and fixed and variable cost. We assessed the CDSS in terms of its financial and economic cost implications. We also conducted a sensitivity analysis on the estimations.

**Results:**

Total financial cost of CDSS intervention amounted to 185,927.78 USD. 77% of these costs were incurred in the installation phase and included all the activities in preparation for the actual operation of the system for client care. Generally, training made the largest share of costs (33% of total cost and more than half of the recurrent cost) followed by CDSS software- 32% of total cost. There was a difference of 31.4% between the economic and financial costs. 92.5% of economic costs were fixed costs consisting of inputs whose costs do not vary with the volume of activity within a given range. Economic cost per CDSS contact was 52.7 USD but sensitive to discount rate, asset useful life and input cost variations.

**Conclusions:**

Our study presents financial and economic cost estimates of installing and operating an electronic CDSS for maternal health care in six rural health centres. From these findings one can understand exactly what goes into a similar investment and thus determine sorts of input modification needed to fit their context.

**Electronic supplementary material:**

The online version of this article (doi:10.1186/s12913-015-0780-9) contains supplementary material, which is available to authorized users.

## Background

Burden of maternal and newborn diseases is still high globally and particularly in sub-Saharan Africa (SSA) [1]. In Tanzania, maternal mortality, neonatal mortality and infant mortality stand at 454/100,000 live births, 26/1,000 live births and 51/1,000 live births respectively [2]. Poor quality of health care is among the causes of this high burden. Potential reason for the poor quality is the existence of a “know-do gap” in these settings whereby health workers do not perform to the best of their knowledge [3].

An electronic clinical decision support system (CDSS) was piloted in six rural primary health centers in Tanzania to aid in making correct decisions during routine antenatal care (ANC) and childbirth (reduce the “know-do gap”). A baseline quality assessment in these facilities revealed some weaknesses in maternal care [4], poor accessibility of guidelines was common [5] hampering quality care and a potential reason for maternal mortalities and morbidities in these areas. Clinical decision support system (CDSS) is a potentially beneficial health care intervention [3,6-8]. It can improve quality of maternal and newborn health by aiding effective decisions [3]. Electronic CDSS have been used in developed countries and recently been applied in developing countries [8].

Since no similar projects existed prior to the CDSS at the sites, the program was envisioned to improve performance of health workers by facilitating adherence to WHO guidelines and ultimately improve quality of maternal health care services [3]. Economic assessment of a new technology such as the CDSS is important in ensuring evidence based resource allocation decisions by providing information on intervention costs and benefits [9]. It is important therefore to know the cost implications of this intervention for planning purposes.

Rising health care costs, resource constraints, competing development priorities and high burden of disease facing developing countries have obliged high quality, effective and efficient health interventions. Resources dedicated to less effective and less efficient medical practices represent wasted resources, that otherwise could be allocated to more effective and efficient health practices, thereby reducing public welfare [9]. This study was consequently an assessment of resources used in installing and operating an electronic CDSS for maternal and newborn care (MNC) and the costs associated with those resources in the six rural health centers. Therefore, in the next sections we will be discussing a cost analysis of the CDSS intervention.

## Methods

### Description of the CDSS program

CDSS is stand-alone, java-based software piloted by the QUALMAT project, a collaborative project funded by the European Union, aimed at improving the quality of maternal and newborn care in selected rural health facilities of Tanzania, Ghana and Burkina Faso. The CDSS was developed after an assessment of quality of maternal health care in the study sites [4]. It incorporates knowledge from the World Health Organization (WHO) guideline “Pregnancy, Childbirth, Postpartum and Newborn Care; A Guide for Essential Practice” with adjustments to fit the local context [3].

Medical experts, users and IT specialists jointly developed the CDSS. To ensure high quality, the process involved 3 iterative test phases that enabled development of a final software version. The CDSS provides computerized guidance and clinical decision support for routine antenatal care and care during delivery and up to 24 hours post-delivery: (1) It provides guidance through routine actions in maternal and perinatal care- supports complete history-taking, physical examination, basic laboratory tests, as well as provision of counseling and preventive measures; (2) It integrates clinical data to detect situations of concern by algorithms- it screens entered data and suggest diagnoses or alert the user about dangerous situations that require consideration during the visit; and (3) It provides electronic tracking of peri- and post natal activities, using an electronic partograph, which provides continuous monitoring of the delivery process on the screen.

In Tanzania, the system was piloted in six health care centres located in rural Lindi District Council. To enable CDSS operation, each study site was equipped with one laptop containing the CDSS software. The study sites did not have computers prior to this study because all routine services were paper based. The system was used for routine antenatal and childbirth care but did not replace the existing paper based system; at the end of each consultation the health workers would complete the usual paper work. To ensure sustainability the computers were standard in terms of affordability, use and maintenance. The laptops were password protected; only health workers trained on CDSS operation were allowed access. Further, infrastructure at the sites supported computer operation; all sites had sustainable power supply from the main grid and/or solar power.

Basic computer skills together with specific knowledge required for safe handling of CDSS hard- and software and the collected data was provided to health workers prior to use of CDSS for MNC services. IT specialist provided technical assistance and supportive supervision regularly to ensure consistency in CDSS use. CDSS design process started in June 2009 but official use for MNC services started in April 2012.

### Study setting

The six health care centres (five public and one private), where CDSS was piloted, are located in rural Lindi District Council. Lindi District Council is within Lindi region in southern Tanzania covering 7,538 sq. km of land. It has 194,143 people- 91,647 males and 102,496 females [10]. The district is predominantly rural with most of its households below the poverty line [11]. Households average around 3.7 members, with subsistence agriculture as the main economic activity.

The district has 1 hospital, 6 health centers and 38 dispensaries in total. Comprehensive obstetric services for Lindi rural are offered by Sokoine hospital located in Lindi town. The number and quality of human resource for health is a big challenge in these rural areas, resulting to poor quality of MNC and high maternal and neonatal disease burden, morbidity and mortality wise [12]. A baseline research conducted in 2010 in the study facilities identified some gaps in the quality of maternal and newborn care [4]. The QUALMAT project therefore aimed at improving the quality of maternal and newborn care by the means of improved health worker’s performance using CDSS as one of the interventions [3]. The CDSS was tailored to fit the Tanzanian context.

### Identification, valuation and calculation of cost

This study was conducted retrospectively from the program perspective. Data were retrieved from accounts records using unstructured questionnaire and interviews with project personnel. Data collection was done between June and August 2013. CDSS intervention costs were estimated using Ingredients approach- a micro-costing method, as opposed to Expenditure approach- a macro-costing method. The Ingredients approach, though more costly to conduct in terms of time and resources, was more suitable to our objective of providing precise estimates of exactly what went into a CDSS intervention, a very useful information for replication to other settings [13,14].

Ingredients approach accounts for all the costs incurred by an intervention. Three distinct phases are involved in this approach: (i) Identification of ingredients required for an intervention, this step requires an understanding of the intervention and all inputs that go into it; (ii) Determination of the value or cost of the ingredients and the overall cost of an intervention. In doing this, all ingredients are assumed to have a cost regardless of source, whether purchased or donated. Usually market prices are used in valuation and where they do not exist, shadow prices are used instead, and (iii) Analysis of the cost in an appropriate decision-oriented framework depending on the objective of the analysis [13].

We broadly grouped cost of CDSS intervention into two categories, installation cost and operation cost. Installation cost category comprised of resources expended before the actual CDSS operation, the time between the decision to implement CDSS and the start of actual operation to the first beneficiary (June 2009-March 2012). Operation cost category comprised of resources expended during actual use of CDSS for MNC services to clients (April 2012- March 2013).

The cost categories consisted of the following inputs: (i) Capital cost – the one-time investments- included the CDSS software, a motor vehicle, computers, furniture and facility (space and electricity), and (ii) Recurrent cost – the on-going investments- included transport, personnel (salaries and allowances), short-term computer and CDSS training, supplies and communication (Table 1).

**Table 1:** **Summary table indicating the rationale for inclusion of different cost items**

**Cost category**	**Cost item and inclusion criteria**
1. Capital cost	In this category we included the following;
	a. Laptop computers- used to operate the CDSS.
	b. Furniture- table and chair for CDSS workstation.
	c. Vehicle- for effective support supervision and monitoring (due to distance and terrain, purchase was cheaper as opposed to renting).
	d. Facility- space and electricity for CDSS operation.
	e. CDSS software – estimated market price.
2. Recurrent cost	In this category we included the following;
	a. Transport- running and maintenance cost of the vehicle, and bus, flight and taxi charges.
	b. Personnel- time compensation for personnel including an estimation of health workers cost.
	c. Training- all costs incurred during basic computer skills and CDSS specific skills training (training materials, allowances, transport, venue and refreshments).
	d. Supplies- office supplies and consumables (stationery, printing, photocopying etc.).
	e. Communication- mainly internet and telephone cost.

The CDSS was developed in response to the quality gaps discovered at the sites as explained above, the CDSS was envisioned to improve health workers’ performance by ensuring that the health workers approached care according to WHO guidelines in providing MNC services to clients. It was not possible to get an accurate estimation of the costs that went into the development of the CDSS because there was no reliable record of resource use and payments; therefore we had to make an estimation of its market price.

There is a scarcity of evidence on health information technology (HIT) software market prices. A few sources have reported a range of HIT software prices, between 4,000 USD and 30,000 USD [15-17]. None of the software programs were directly comparable to our CDSS. Moreover, some of these figures included costs over and above the software itself such as training and support components. Thus we estimated a market price for the CDSS at 10,000 USD per health center for our case as this is exclusive of all other costs such as training and support. Uncertainty in the price estimation was taken care of in sensitivity analysis. The CDSS is a technological non-physical capital asset.

It was necessary to purchase a motor vehicle due to several reasons. First, all intervention sites are more than 400 kilometers from Muhimbili University where the project was based. For monitoring and supportive supervision, personnel based in Lindi would routinely travel between 30 to 100 kilometers to project sites, while those based in Muhimbili University would travel an additional 400 kilometers. Second, the roads to these rural sites were not reliable especially during rainy seasons. These made it necessary to purchase a suburban utility vehicle (SUV), a more useful vehicle for such state of affairs. Purchase as opposed to renting was a less expensive option.

The vehicle was used in several activities, including evaluation research of the QUALMAT project at the 6 intervention sites (Lindi health facilities under study) and 6 control sites (Mtwara health facilities) and health worker motivation research. CDSS related activities that made use of the car included training, meetings with local health officials, supportive supervision and monitoring of the intervention. Usually in a day the car would be used exclusively for specific project activities. The vehicle would be used the whole day in the field (morning to evening) and then parked afterwards. Because of the cost (from travel distance, fuel, personnel and time to and from the sites) all planned activities were accomplished within the particular day which left no vehicle time for other unplanned non-project purposes.

Cost of vehicle for CDSS installation and operation is the proportion of the total vehicle time spent on CDSS. Specifically, during the project period we estimated that the vehicle was in full use around 527 days for the above named activities and out of those 261 days were solely for CDSS activities, we did not consider inactive days. Therefore we estimated CDSS activities used the car 49.5% of the time – 261 divided by 527 multiplied by 100. We multiplied 49.5% by the vehicle purchase price to get vehicle cost for CDSS installation and operation.

A total of 7 laptop computers were purchased for the intervention, one laptop for each health center and 1 for backup. These computers were exclusively used for CDSS. Also a set of furniture, a table and chair was purchased for CDSS use. Facility (included space and electricity) was donated by the health centers, QUALMAT did not pay for these, nonetheless for the purposes of estimating economic cost of the intervention, we estimated facility cost using market price per square meter including electricity.

All capital inputs were annualized. Annualization gives equivalent annual cost of the capital items, where capital costs are spread over the useful life of the input [18-20]. We used the following annuitization formula by Drummond et al. [19].

$$ K=\frac{E}{\left[1+r\right]}+\frac{E}{{\left[1+r\right]}^2}+\cdots \cdots +\frac{E}{{\left[1+r\right]}^n} $$

Where; *K* is capital outlay*, E* is equivalent annual cost*, r* is discount rate and *n* is number of useful years for the capital item [19]. The CDSS was annualized using a 3% discount rate and 10 years useful life, the vehicle was annualized using a 3% discount rate and 10 years useful life, equipment was annualized using a 3% discount rate and 5 years useful life and facility was annualized using a 3% discount rate and 30 years useful life.

Transport cost comprised vehicle operation and maintenance costs, and other travel costs including taxi, bus and flight fares. To keep the vehicle running, costs of routine repairs, insurance, battery, tires and fuel were incurred during the intervention. Included costs are only for CDSS related activities.

Personnel costs included time compensation (salaries and allowances) for people who were involved in the CDSS intervention. One project administrator was responsible for day to day running of the project specifically monitoring, supportive supervision and handling CDSS database, was paid a monthly salary of about 597 USD and during trainings she was paid daily allowances of about 48 USD. One IT specialist was responsible for training health workers on how to operate CDSS, maintenance of the system including upgrade of software and providing IT support for CDSS software and hardware and also supportive supervision, was paid around 48 USD per day for the duration of a specific activity. Two gynecologists and one behavioral scientist were involved in monitoring and supportive supervision, they were also paid around 48 USD per day for the duration of a specific activity. A driver of the project vehicle was paid around 358 USD per month.

QUALMAT Tanzania did not pay allowances to health workers for CDSS use in client care because the system was integrated into the routine maternal care, however for the purpose of economic cost assessment it was assumed a health worker would be paid 5,000 Tanzanian Shillings (approximately 3 USD) per ANC contact and 10,000 Tanzanian Shillings (approximately 6 USD) per childbirth using CDSS. We did not include routine health workers costs (salaries, benefits etc.) as these were borne by the government.

Three rounds of trainings were conducted. Basic computer was taught to 38 health workers then CDSS specific training on software and hardware use and maintenance to 43 participants including health workers and council health management teams (CHMT). Refresher training was later offered to 30 health workers to ensure competency of CDSS use among the health workers and to take care of health worker turn-over. Training involved costs of material production; per diem and travel allowances to participants and facilitators; venue; equipment; stationery; visual aids and refreshments such as food and drinks.

Other intervention costs included office supplies- comprising mostly of stationery, and communication- mobile phones, teleconference and internet.

We estimated financial costs and economic costs separately. Financial costs are the real-money outlays for resources required to produce an intervention and to manage a patient’s health outcome while economic costs of an intervention are the opportunity costs of the resources used to implement the intervention [20]. Both provide useful information for decision makers, financial costs show the actual money spent while economic costs in addition show the value of inputs for which no money was spent, capture the cost of a good when the price does not reflect the cost of using it productively elsewhere and show equivalent annual cost of capital items; in this regard economic costs give a more complete picture of resource use [21].

In financial cost analysis therefore the costs reflected how much and when the money was spent during CDSS intervention by QUALMAT, while in economic cost analysis the costs in addition reflected the cost of donated goods and services (health workers time and facility cost) used and the equivalent annual cost of capital items. To get total costs, quantities of inputs used in the intervention were multiplied by their respective unit prices.

The economic costs were further categorized into fixed costs (costs that do not change with output level) and variable costs (costs that change proportionately with output level). CDSS cost per MNC contact was calculated by dividing total economic cost of CDSS intervention by the total number of MNC contacts using CDSS.

A multivariate sensitivity analysis was conducted to account for varying discount rate, useful life of capital items and input cost. Specifically we considered 3%, 5% and 10% discount rates; varied useful life for CDSS software to 5 years and 15 years, varied useful life for vehicle to 5 years and 12 years, varied useful life for computers and furniture to 3 years and 7 years, varied useful life for facilities to 20 years and 40 years; and adjusted all cost variables by a 20% markup or markdown and discussed the implications on the economic cost of CDSS intervention and explicitly on cost per MNC contact using CDSS.

Costs in local currency (Tanzanian Shillings- TZS) were converted into United States Dollars (USD) using European Union exchange rates prevailing at the time of expenditure [22]. The exchange rates ranged between 1 USD = 1410 TZS to 1 USD = 1676 TZS in the period 2010 and 2013 [23]. Excel spreadsheet was used for data analysis.

### Ethics

The Muhimbili University of Health and Allied Sciences (MUHAS) ethics committee (reference number MU/RP/AEC/Vol.XIII/1) and the ethics committee of the University of Heidelberg (reference number S-173/2008) approved the study. Informed consent was obtained from participants.

## Results

### Financial cost of CDSS intervention

Total financial cost of CDSS intervention in all the six health centers amounted to 185,927.78 USD (Table 2), equivalent to 30,987.96 USD per health center. 77.2% of these costs were incurred during the installation phase (covering period June 2009-March 2012), which included all activities in preparation for the actual use of the CDSS for client care. In this phase a large investment in capital inputs and training was done. The operation phase (April 2012- March 2013) used only 22.8% of the total financial costs.

**Table 2:** **Financial cost of CDSS intervention in USD, 2009-13**

**Cost category and items**	**Installation cost (2009–12)**	**Operation cost (2012–13)**	**Total cost**	**Percentage of total cost**
**Recurrent cost**				
Transport			12243.02	6.6
*Vehicle operation and maintenance*	*5743.30*	*5411.88*	*11155.19*	
*Travel*	*529.03*	*558.80*	*1087.83*	
Personnel	5767.73	12579.66	18347.39	9.9
Training	38665.09	22777.60	61442.68	33.0
Other			3,019.69	1.6
*Supplies*	*1594.83*	*569.44*	*2164.27*	
*Communication*	*855.43*	*-*	*855.43*	
**Sub-total: recurrent cost**	**53155.40**	**41897.39**	**95052.79**	**51.1**
**Capital cost**				
Vehicle	22571.13	-	22571.13	12.1
Computers	7865.84	-	7865.84	4.2
Furniture	-	438.03	438.03	0.2
CDSS software	60000.00	-	60000.00	32.3
**Sub-total: capital cost**	**90436.96**	**438.03**	**90874.99**	**48.9**
**Total cost**	**143592.36**	**42335.42**	**185927.78**	**100**
**Percentage of total cost**	**77.2**	**22.8**		**100**

Total capital costs (for both phases) made up about half (48.9%) of total financial cost of which the largest portion (32.3%) was cost of the CDSS software, 12.1% was the cost of one project vehicle, 4.2% was the cost of seven laptop computers (six laptops for the sites and one for backup) and 0.2% cost of furniture for CDSS workstation (Table 2).

51.1% of total financial costs (for both phases) were recurrent (Table 2). Training made the largest share (33% of total financial cost and more than half of the recurrent cost) followed by personnel which took 9.9% of total financial cost, transport consumed 6.6% while 1.6% were other costs.

### Economic cost of CDSS intervention

In estimating economic cost, we went a step farther and included estimate of the donated health workers’ time and facility (space and electricity), and also annualized capital costs as explained in the methods section. Total economic cost of CDSS intervention in all the six health centres amounted to 127,506.17 USD, equivalent to 21,251.03 USD per health center, a difference of 31.4% from the total financial cost above. Installation cost made 50.6% of the total economic cost; the rest were incurred during operation (Table 3).

**Table 3:** **Economic cost of CDSS intervention in USD, 2009-13**

**Cost category and items**	**Installation cost (2009–12)**	**Operation cost (2012–13)**	**Total cost**	**Percentage of total cost**
**Recurrent Cost**				
Transport			12243.02	9.6
*Vehicle operation and maintenance*	*5743.30*	*5411.88*	*11155.19*	
*Travel*	*529.03*	*558.80*	*1087.83*	
Personnel	5767.73	22045.65	27813.38	21.8
Training	38665.09	22777.60	61442.68	48.2
Other			3019.69	2.4
*Supplies*	*1594.83*	*569.44*	*2164.27*	
*Communication*	*855.43*	*-*	*855.43*	
**Sub-total: recurrent cost**	**53155.40**	**51363.38**	**104518.78**	**82.0**
**Capital cost**				
Vehicle	2646.03	2646.03	5292.05	4.2
Computers	1717.54	1717.54	3435.09	2.7
Furniture	-	95.65	95.65	0.1
Facility (space and electricity)	-	96.94	96.94	0.1
CDSS software	7033.83	7033.83	14067.67	11.0
**Sub-total: capital cost**	**11397.40**	**11589.98**	**22987.39**	**18.0**
**Total cost**	**64552.81**	**62953.36**	**127506.17**	**100**
**Percentage of total cost**	**50.6**	**49.4**		**100**

Capital costs were smaller after annualization, made about 18% (as opposed to the original 48.9% in the financial cost results) of total economic cost (Table 3). CDSS software, vehicle, computers, furniture and facilities made 11%, 4.2%, 2.7%, 0.1% and 0.1% of total economic cost respectively.

Recurrent costs made 82% of total economic cost. Distribution of specific recurrent costs relative to total economic cost showed similar trend to the financial cost results but slightly higher percentages, training made 48.2%, personnel made 21.8%, transport made 9.6% of total economic cost while 2.4% were other costs.

### Fixed costs, variable costs and average costs

Fixed costs made up more than three quarters of the total economic cost (Table 4). Variable costs for CDSS intervention included health workers’ time compensation paid per MNC contact and cost of electricity used by CDSS operation. Fixed costs were 117,991.71 USD or 92.5% while variable costs were only 9,514.46 USD or 7.5% (Table 4).

**Table 4:** **Fixed, variable and average costs of CDSS intervention in USD, 2009-13**

**Item**	**Total**	**Percentage**
Fixed Cost (CDSS)	**117991.71**	**92.5**
Variable Cost (CDSS)	**9514.46**	**7.5**
Total Economic Cost (CDSS)	**127506.17**	**100.0**
Total ANC contacts registered at study sites (1 year)	**2386.00**	**100.0**
Total ANC contacts using CDSS (1 year)	**1665.00**	**70.0**
Total childbirths registered at study sites (1 year)	**887.00**	**100.0**
Total childbirths using CDSS (1 year)	**754.00**	**85.0**
Total CDSS contacts (ANC plus childbirths using CDSS)	**2419.00**	
Cost per CDSS contact (Total Economic Cost (CDSS)/Total CDSS contacts)	**52.71**	

During the one year period of CDSS operation 2,386 ANC contacts were recorded in the health centers, of these 1,665 (70%) ANC contacts were registered in the CDSS; and 887 childbirths were recorded in the health centres, of these 754 (85%) were registered in the CDSS. Total MNC contacts registered in the CDSS were therefore 2,419 and therefore economic cost per CDSS contact was 52.71 USD (Table 4).

### Sensitivity analysis

**Figure 1:**
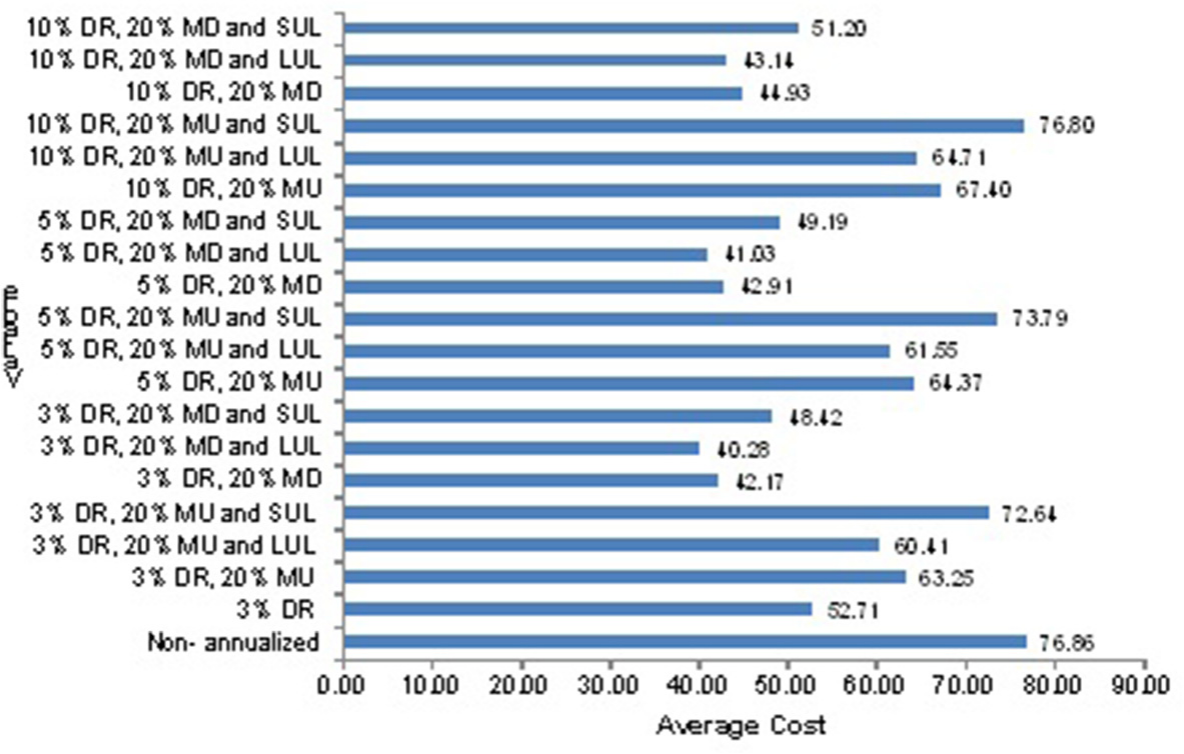
**Sensitivity analysis on economic cost of CDSS intervention.** This figure displays results from a multivariate sensitivity analysis where discount rate, useful life and input cost were varied. DR = Discount rate, MU = Mark-up and MD = Mark down.

Results from the multivariate sensitivity analysis showed that our estimates are sensitive to assumptions made about cost of inputs, discount rate and capital asset useful life (see Additional file 1). When the cost of inputs, discount rate and capital asset useful life were varied, total economic cost of CDSS intervention ranged between 97,426.73 USD and 185,769.43 USD, and economic cost per CDSS contact ranged between 40.3 USD to 76.8 USD (Figure 1).

## Discussion

This paper presents an assessment of the cost of installing and operating an electronic CDSS for routine maternal health care in six health centers located in rural resource-poor settings of Tanzania. Results show total financial cost of the intervention was 185,927.78 USD while total economic cost of the intervention was 127,506.17 USD. Most financial costs were incurred in the installation phase as expected; this phase took around two years and engaged heavy investment in CDSS training and capital inputs. Other cost studies have also reported high intervention installation cost [24,25].

Training is the most important activity of any CDSS intervention, health workers need to be well trained for a successful health technology intervention [26]. Training was central in both phases of the intervention as witnessed by the high training cost, which is more than quarter of the financial cost and close to half of the economic cost. The majority of health workers lacked basic computer knowledge and skills consequently more training cost was incurred in the installation phase involving basic training on computer use and CDSS specific training. Because of personnel turnover (some personnel transferred to other health facilities or went for studies) subsequent training was necessary. Training has been found to take up substantial resources in other CDSS interventions as well [27].

Cost studies have demonstrated the importance of personnel to any health intervention [25,27,28], this is true for CDSS intervention as well. Our results show moderate personnel cost. Project personnel were vital to providing training and supportive supervision to health workers, while health workers were important in making sure the CDSS was utilized for client care. Most personnel costs were incurred during the operational phase of CDSS. It was necessary to provide constant supportive supervision to health workers in order to ensure right use and consistency. Payment to compensate health workers’ time is essential to motivate CDSS utilization, especially at the outset.

As health workers become conversant and the CDSS is integrated into the routine system these costs may fall as it may not be necessary to remunerate CDSS use (apart from their usual salaries and benefits) and trainings and supportive supervisions will be less frequent. Given the influence of health worker training on the success of CDSS, the problem of health worker retention facing most rural health care centres in developing countries may be a challenge to CDSS use especially at the beginning before total integration of the system.

From our results, on average it will cost about 21,251.03 USD per health center to adopt the CDSS in a similar setting. These figures reflect the cost of installing the CDSS and operating it for one year. As most inputs for CDSS intervention are fixed it means that marginal cost of CDSS would be very small promising economies of scale with increased activity level (CDSS contacts). It is beneficial to remunerate health workers’ use of the CDSS in order to motivate utilization.

Our results show the value of the information that can be derived from estimating economic costs in addition to the conventional financial costs in a costing exercise. In financial analysis, almost all capital costs were incurred in the installation phase while in economic analysis capital costs were almost evenly distributed between the two phases. Equivalent annual costs of capital inputs gave a clearer picture of the use of capital items during their useful life. Not doing so over-estimates the actual magnitude of resource use in its first year while underestimating its use in subsequent years thus leading to biased estimates of costs.

Furthermore, donated goods and services have opportunity costs which are important to capture in understanding the true value of investments to society. This is especially true in making decisions regarding the CDSS in contexts where donated goods and services may have to be paid for. Financial cost estimation does not take into account opportunity cost of donated resources as it only reflects monetary transactions and thus does not give a full evaluation of resource use. To this end economic cost estimations supplement financial cost estimations.

From this study interested parties can get cost information to apply in their contexts; specifically, a decision maker can use the results to budget for the CDSS, make judgments whether the CDSS will be affordable to adopt and cost implications of scaling-up and if the intervention will be sustainable according to local capacity. Financial cost information is most useful for budgeting while economic cost information is more comprehensive. These results can be used together with CDSS effectiveness information to conduct a full economic evaluation of CDSS to ascertain its efficiency.

Once the CDSS is proven to be efficient, its adoption and replication will primarily require investment in the CDSS software, sustainable equipment, power, training (including supportive supervision), and some remuneration to motivate CDSS use especially at the beginning. Other likely future costs are system updates which may cost around 1000–1500 USD and maintenance costs. The current move by the Tanzanian government to use HIT to improve health care quality, efficiency and equity [29] is a good sign for the future of technologies such as the CDSS under study.

Sensitivity analysis results show CDSS intervention costs are sensitive to the input price, discount rate and the length at which capital assets are used. In poor settings such as the ones under study where resources are limited, investing in capital assets of high quality and making use of them to capacity is necessary.

Moreover, with the advent of handheld devices that are cheaper compared to desktop and laptop computers, and the rapid technological advancement that promises more durable, versatile and cheap devices; CDSS intervention cost may go down in the future. This together with the possible cost saving benefits of CDSS use, in terms of increased MNC care quality and improved population health outcomes, may cut cost of health care in the long run. Handheld devices have been shown to be effective in supporting clinical decision making at the point of care [30,31].

One similar study in Ghana, where the QUALMAT CDSS (adapted to that context) was implemented, estimated financial cost of implementation was 23,316 USD while economic cost was 17,128 USD [27]. The huge gap between these two studies is due to the type and cost of inputs in the analysis which is mainly driven by contextual differences between the two countries. Specifically, the study did not consider CDSS software cost; had lower transport and travel cost (no vehicle purchased, their sites are located at a close proximity); had lower personnel costs because of differences in remunerations and working environment; and had lower training costs because of the way the trainings were designed and conducted.

We were not able to find other directly comparable studies with which to make meaningful comparison of our results, there are a number of HIT costing studies in other countries with varying cost estimates depending on the type of technology, parameters used and analysis assumptions- the costs range from thousands to millions of US Dollars [28,32-35].

### Study limitations

At this stage we can only provide a cost analysis of the system, however a full economic evaluation is scheduled; cost-effectiveness analysis of the CDSS will be a more comprehensive study of efficiency of the investment made.

## Conclusions

Our findings show information on CDSS installation and operation cost in rural health centres. From these findings one can understand exactly what goes into a similar investment and thus determine sorts of input modification needed to fit their context. Interested parties may use the financial and economic cost information to apply in their contexts; specifically, a decision maker can use the results to budget for the CDSS, make judgments whether the CDSS will be affordable to adopt and cost implications of scaling-up and whether the intervention will be sustainable according to local capacity. Findings suggest cost of adopting and scaling-up the system is context dependent. Retention of health workers will be an important factor for the success of CDSS. To give a more comprehensive analysis of CDSS efficiency a full cost-effectiveness analysis is necessary.

## Additional file

Additional file 1:
**Sensitivity analysis results.** This table displays results from a multivariate sensitivity analysis where discount rate, useful life and input cost were varied.

## Abbreviations

ANC: Antenatal care
CDSS: Clinical Decision Support System
HIT: Health Information Technology
Sq. km: Square kilometer
TZS: Tanzanian Shillings
USD: United States Dollars
WHO: World Health Organization
